# Association of grip strength and gait speed with structural brain volume in older adults with and without cognitive impairment.

**DOI:** 10.1002/alz.091788

**Published:** 2025-01-03

**Authors:** Keith R Cole, Kelsie M Full, Hui Shi, Kimberly R. Pechman, Niranjana Shashikumar, L. Taylor Davis, Timothy J. Hohman, Angela L. Jefferson

**Affiliations:** ^1^ Vanderbilt University Medical Center, Nashville, TN USA; ^2^ Division of Epidemiology, Department of Medicine, Vanderbilt University Medical Center, Nashville, TN USA; ^3^ Vanderbilt Memory and Alzheimer’s Center, Vanderbilt University Medical Center, Nashville, TN USA; ^4^ Vanderbilt Memory & Alzheimer’s Center, Vanderbilt University Medical Center, Nashville, TN USA

## Abstract

**Background:**

Growing evidence supports that measures of overall physical health are related to incidence and progression of Alzheimer’s disease and related dementias (ADRD). In particular, measures of gait speed and grip strength independently predict incident dementia and cognitive decline in older individuals. Less is known regarding their relationship with structural brain volume in the presence of cognitive impairment. This study examined the relationship between gait speed and grip strength, and regional brain volumes in older adults with and without cognitive impairment.

**Methods:**

Participants included 570 adults (59% males; n = 412 cognitively unimpaired; n = 158 mild cognitive impairment) aged 50‐92 years (M = 69.0, SD = 9.0) from the Vanderbilt Memory and Aging Project. Participants underwent 3T brain magnetic resonance imaging (MRI) at study enrollment. Cross‐sectional multivariate linear regression models were used to determine the associations between gait speed (usual walking pace over 15 ft) and grip strength (greatest of three maximum effort trials) with hippocampus and frontal, temporal, parietal, and occipital lobe volumes. Covariates included age, biological sex, ethnicity, education, BMI, global depression scale, Framingham Stroke Risk Profile, APOE ε4 status, cognitive status, and intracranial volume. The model included interaction terms of gait speed and grip strength each with biological sex and cognitive status.

**Results:**

In fully adjusted models, poorer grip strength was only associated with lower hippocampal volume (F(13,491) = 26.3, R^2^_adjusted_ = 0.4, p = 0.001). Sex interacted with grip strength (p = 0.03) on hippocampal volume such that the association was present among male (p = 0.006) but not female participants (p = 0.46). Slower gait speed was only associated smaller frontal lobe volume (F(12,488) = 51.0, R^2^_adjusted_ = 0.56, p = 0.04) without interaction with sex (p = 0.95). There was no evidence of interaction with cognitive status in any model (all p’s>0.05).

**Conclusion:**

Grip strength and gait speed, both quick and easy clinical measures, are differentially related to structural brain volume. Grip strength is related to hippocampal volume only in male older adults with and without cognitive impairment, indicating potential influence of biological sex. Gait speed, however, is related to frontal lobe volume in both males and females. Changes in distinct measures of physical health of those with and without cognitive impairment may indicate changes in different regions of the brain.

